# Biochemical assessment in a cohort of pediatric patients with cystic fibrosis

**DOI:** 10.25122/jml-2024-0288

**Published:** 2024-06

**Authors:** Corina-Elena Anghel (Delia), Andreea-Mariana Negrescu, Iustina-Violeta Stan, Simona Raluca Iacoban, Elena-Simona Moiceanu, Geanina-Mirela Toma, Elena Ionica, Anisoara Cimpean

**Affiliations:** 1 Faculty of Biology, University of Bucharest, Bucharest, Romania; 2 Alessandrescu-Rusescu National Institute for Mother and Child Health, Bucharest, Romania; 3 Faculty of Medicine, Carol Davila University of Medicine and Pharmacy, Bucharest, Romania; 4 Faculty of Medicine, Iuliu Hațieganu University of Medicine and Pharmacy, Cluj-Napoca, Cluj-Napoca, Romania; 5 Emergency Clinical Hospital for Children 'Grigore Alexandrescu'

**Keywords:** cystic fibrosis, nutritional status, fat-soluble vitamins, biochemical assessment

## Abstract

Cystic fibrosis (CF) is a recessive inherited disorder caused by genetic mutations in the CF transmembrane conductance regulator (*CFTR*) gene. It is a multisystem condition that primarily induces abnormal mucus accumulation in the respiratory system and obstructs the intrapancreatic common bile duct, causing a reduction in the delivery of digestive enzymes to the small intestine. Thus, patients with CF are characterized by maldigestion, malabsorption, and recurrent airway bacterial infections. Clinical monitoring of the health status of patients with CF is mandatory for increasing the patients’ lifespan. To assess the feasibility of monitoring life quality (LQ) in pediatric patients with cystic fibrosis (CF) and to explore the relationship between biochemical parameters and clinical symptoms, our study analyzed inflammatory responses related to CF, medication, and pulmonary bacterial infections in 52 patients diagnosed with CF. Blood, hypo-pharyngeal exudate, and fecal samples were analyzed using clinical biochemistry, hematology, and microbiology techniques at the Alessandrescu-Rusescu National Institute for Mother and Child Health central laboratory in Bucharest, Romania. All the participants adhered to their prescribed outpatient CF regimens and appeared clinically stable. The overall clinical status of patients with CF was observed and compared with that of a healthy control group, which consisted of individuals similar in number and age. The screened patients with CF presented an impaired lipid status and chronic infections with various bacteria, iron, and vitamin (A, D, and E) deficiencies. Our findings provide insights into the pathophysiological mechanisms of CF and suggest that tailored monitoring and personalized therapeutic strategies could improve patient management.

## INTRODUCTION

With a rate of occurrence of one in every 2,500 live births, cystic fibrosis (CF) is one of the most common life-threatening autosomal recessive disorders, with a higher prevalence among individuals of European descent [1]. The main pathology of CF is represented by an excessive build-up of abnormally viscous secretions at the luminal surface of the epithelial cells found in various important organs such as the lungs, pancreas, and gut, which in time leads to obstructions, infections, intense inflammatory activity, and organ failure [2]. After 1989, it became universally acknowledged that the underlying cause of CF is represented by genetic mutations in the cystic fibrosis transmembrane conductance regulator (*CFTR*) gene located on chromosome 7q31.2 [3], which encodes the *CFTR* protein responsible for the epithelial chloride ion channel, a focal point of modulation for several important functions [4]. Mutations in *CFTR* result in reduced or absent protein function, affecting several physiological processes [5]. Since the early 2000s, approximately 2,000 *CFTR* gene variants have been documented in the Cystic Fibrosis Mutation Database, although the full functional and pathophysiological implications of these mutations are not yet completely understood [6]. When the functional effects of these mutations are known, they are categorized into one of seven established classes.

In the respiratory system, the abnormal mucus adheres to the surface of the respiratory tract, leading to a decrease in mucociliary clearance and a higher risk of infection and chronic inflammation. In the pancreas, the build-up mucus obstructs the intrapancreatic common bile duct, reducing the delivery of digestive enzymes to the small intestine and impairing nutrient absorption [7,8]. This malabsorption, combined with the high metabolic demands of maintaining homeostasis, leads to poor nutritional intake, malnutrition, and deficiencies in vitamins and minerals [9,10]. Thus, *CFTR* mutations can cause a range of issues affecting multiple organs with varying severity.

Nutritional deficiencies in patients with CF are influenced by several factors. The most important are *i)* pancreatic insufficiency, which leads to fat, protein, and micronutrient malabsorption; *ii)* bile salt disturbance, which leads to fat vitamin malabsorption; *iii)* chronic inflammation that is associated with increased activity of the apical calcium channels, leading to an elevated local calcium concentration; *iv)* heightened energy needs due to impaired lung function; *v)* chronic bronchopulmonary obstruction and bacterial infection with biofilm forming gram-negative organisms, especially *Pseudomonas aeruginosa* and recurrent lung infection; and *vi)* decreased nutrient intake, especially during periods of acute illness [11]. While fat-soluble vitamin deficiencies in CF patients have been well-studied, the impact of mineral and trace element depletion, especially during acute exacerbations, remains less understood [11]. Thus, as an integral part of the multidisciplinary care of patients with CF, nutrition management approaches have included anthropometric assessment (typically measurements of body mass index [BMI] and body weight over time), monitoring levels of specific nutrients like vitamin D, zinc, and vitamin A, and various interventions including oral caloric and protein supplementation, pancreatic enzyme replacement therapy, appetite stimulants, enteral tube feeding, and micronutrient supplementation [9,12,13].

The European Society of Cystic Fibrosis (ESCF) recommends supporting newborn screening programs to ensure early CF diagnosis and prevent delays [14]. Diagnosis primarily relies on detecting abnormal *CFTR* function through sweat chloride testing, with a threshold of ≥60 mmol/L, alongside positive newborn screening results, clinical features consistent with CF, or a positive family history [1]. The sweat test uses pilocarpine iontophoresis and is considered the gold standard for diagnosing CF. However, limitations and inconsistencies in the sweat test technique have led to numerous false-positive and false-negative results over the years. Additionally, awareness of genetic testing remains low among the general population, and the high costs associated with this diagnostic tool make it inaccessible to many individuals [14].

Clinical analyses of biochemical parameters have long been used to monitor the biochemical status of patients with CF and guide pediatricians in providing personalized treatment [13-16]. In Romania, research has predominantly focused on the progress and quality of life of patients with CF over the age of 18. The pathology is often underdiagnosed due to the limited availability of laboratory tests. A review of literature data about the Romanian prevalence and research on cystic fibrosis pathology indicated only two publications on the prevalence of Ig-E-mediated food allergies and new missense mutations identified in the Romanian CF population. Specific analyses conducted during CF patient monitoring are less accessible due to inadequate equipment or funding from national programs. The national screening for CF was introduced in July 2022 and did not include the assessment of fat-soluble vitamin status and elastase level. Vitamin D status is evaluated under another national health program. Early detection of fat-soluble vitamins A and E deficiencies in these children could improve dietary recommendations and earlier interventions to prevent long-term complications. Approximately 413 pediatric patients with CF are managed across several clinics in Romania, with three located in Bucharest.

In this context, our study aimed to analyze the clinical profiles and fat-soluble vitamin levels of children diagnosed with CF enrolled between 2021 and 2023 at the Bucharest Regional Centrum NIMCH Alessandrescu-Rusescu, using biochemical, hematological, and microbiological assessments. This pilot study will contribute to long-term research on CF in Romania and support the development and regular updating of national guidelines for monitoring and treating pediatric CF patients.

## MATERIAL AND METHODS

### Selection and description of participants

This descriptive, retrospective study included 68 pediatric patients diagnosed with cystic fibrosis and registered at the Bucharest Regional CF Centre of INSMC Alessandrescu-Rusescu between 2021 and 2023. Inclusion criteria required patients to be between 2 months and 18 years old and have a CF diagnosis confirmed by sweat chloride testing (pilocarpine iontophoresis), the gold standard for CF diagnosis. At enrollment, all patients underwent a comprehensive evaluation that included clinical assessments (nutritional status and growth development through anthropometric measurements such as weight in kg, waist/height in cm, BMI in kg/m^2^, and Z-scores for BMI, weight, and height, objective examination on devices and systems) and paraclinical evaluations. Of the initial cohort, 69.66% (*n* = 62) met the eligibility criteria: two positive sweat tests (chloride levels ≥60 mEq/L), a confirmed *CFTR* gene mutation, adherence to dietary and treatment regimens, and regular follow-up visits. Five patients were excluded due to refusal of voluntary participation by their parent/caregiver/legal guardian, and another five were excluded due to compromised biological samples. Thus, the final study group comprised 52 patients with CF (20 boys and 32 girls) who met all inclusion criteria.

All patients with CF received personalized standard therapy, including aerosolized bronchodilators, antibiotics, vitamin supplements, pancreatic enzymes, and high-calorie/protein diets. A control group of 52 healthy subjects (27 girls and 25 boys) was included for comparison. The control group consisted of individuals from similar social classes and ages, of the same ethnicity, with normal biochemical parameters, and who were not on any special medications or nutritional supplements.

### Data collection and measurements

According to the specific protocol for patients with CF, each participant underwent a specialist consultation to monitor their clinical progression, including BMI, height, weight, pulmonary function, pancreatic insufficiency, gastrointestinal disorders, and neuromuscular conditions. Nutritional status was assessed, and blood samples and hypo-pharyngeal exudates were collected. The outpatient regimen for participants included a standard CF diet (Infasource), characterized by high caloric intake supplemented with pancreatic lipase and vitamins A, D, and E, and in some cases, vitamin K.

Molecular testing was conducted at the Regional Centre for Medical Genetics (CRGM) in Bucharest, part of the INSMC 'Alessandrescu-Rusescu' structure, using PCR technology. The center utilized a panel (CF genetic assay nuclear laser medicine genotyping kit from Nuclear Laser Medicine) that included 38 mutations and 1 polymorphism of the *CFTR* gene.

Plasma samples were processed according to the standardized procedures to minimize the risk of contamination. Hemolyzed specimens were discarded to avoid false results. Samples were kept at -20^0^C until assessment. Standard equipment was combined with the corresponding test kits for clinical-chemical analysis. The biochemical parameters, the total number of red blood cells (RBC) and granulocytes, hemoglobin (Hgb), hematocrit (Ht), total protein, uric acid, urea, creatinine, iron, ionized calcium, total calcium, transaminases (aspartate aminotransferase (AST), alanine aminotransferase (ALT)), triglycerides, total cholesterol, and vitamins A, D, and E, were analyzed at the NIMCH Alessandrescu-Rusescu biochemical laboratory. The normal values of biochemical parameters, depending on age and sex, in the Romanian population are presented in Table 1S from Supplementary Material.

**Table 1:** Clinical characteristics of participants

Characteristics	CF patients Median ± SD	Healthy patients Median ± SD
Number	52	52
Age, years	6.60 ± 4.30	4.65 ± 4.23
Gender, % male	57.69%	48.07%
Weight, kg	22.75 ± 3.20	22.99 ± 1.72
Height, cm	112.30 ± 6.36	118.60 ± 3.87
Pulmonary symptoms, %	88.46%	-
% male with pulmonary symptoms)	50%	-
Specific disorders of internal pancreatic secretion, %	67.30%	-
Intestinal malabsorption, %	40,38%	-
Moderate protein-energy malnutrition, %	71.15%	-

Supplementary Material

Hematological parameters were measured using EDTA samples on the XN 1000 instrument (Sysmex Corporation). Clinical chemistry parameters were determined from serum using various diagnostic equipment, including the Dimension EXL™ 200, Vitros ECiQ (Assista), AVL 9180 (Roche Diagnostics GmbH), Cobra Integra 400 Plus (Roche Diagnostics GmbH), VIDAS PC (bioMérieux), and 1290 Infinity II LC System (Agilent). Microbiological analyses of hypo-pharyngeal exudates were performed using the VITEK bacteriology analyzer (bioMérieux). Anthropometric measurements, including BMI, height (cm), and weight (kg), were taken using a portable calibrated stadiometer (Tanita Pro DC430 MA, Tanita Corporation of America), commonly used in clinical research. To account for potential genetic factors (e.g., gender, race) and geo-climatic factors (e.g., sunlight exposure, sunscreen use, diet, direct vitamin D supplementation, skin pigmentation) [16], and to avoid artificially high vitamin D values, vitamin D analysis was conducted during the cold season.

### Statistics

Descriptive analyses were conducted for all variables. For continuous variables, the following statistics were calculated: arithmetic mean, standard deviation (SD), standard error of the mean (SEM), median, and confidence intervals (25th and 75th percentiles). Additional measures included minimum and maximum values, 95% confidence intervals, geometric mean, skewness, and kurtosis. Values reported below the limit of quantification (LOQ) were considered undetected. Data reported below the limit of quantification (LOQ) were considered undetected. GraphPad Prism Software v.9.1.1 was used to manage the study database and perform statistical analyses (San Diego, California, USA). Probability levels with *P* < 0.05 values were considered statistically significant. Data were graphically represented using box plots to illustrate the probability levels and compare the analyzed groups.

## RESULTS

### Groups characteristics

In the current study, we compared the results from 52 patients with CF with those from 52 healthy pediatric subjects. None of the children in this study were diagnosed through newborn screening, as this program was only introduced in Romania in July 2022.

For patients with CF, the *CFTR* variants are detailed in Table 2S of the Supplementary Material. The most common *CFTR* genotype in this group was DF508/DF508, occurring in 46.1% of the patients (Table 3S). At the time of the study, only two of the 52 patients with CF were receiving *CFTR* modulator therapy, largely due to age and financial constraints.

**Table 2:** Comparative liver enzyme levels between patients with CF and healthy controls

Liver biomarker	CF patients Mean ± SD	Healthy patients Mean ± SD	*P* value
AST (UI/L)	33.67 ± 12.71	33.1 ± 15.56	0.81
ALT (UI/L)	36.38 ± 19.82	34.38 ± 16.96	0.57

**Table 3:** Nutritional status across study groups

Nutrients	CF patients Mean ± SD	Healthy patients Mean ± SD	*P* value
Vitamin A (mg/L)	0.29 ± 0.07	0.55 ± 0.15	0.0001
Vitamin E (mg/L)	9.31 ± 4.92	10.1 ± 2.99	0.33
Vitamin D (nmol/L)	61.65 ± 23.94	91.87 ± 34.71	0.0001
Iron (µmol/L)	10.58 ± 5.37	15.29 ± 6.97	0.0002
Ionized calcium (mmol/L)	1.29 ± 0.05	1.3 ± 0.07	0.37
Total calcium (mg/dL)	11.15 ± 2.08	9.64 ± 0.49	0.42

**Figure 1:**
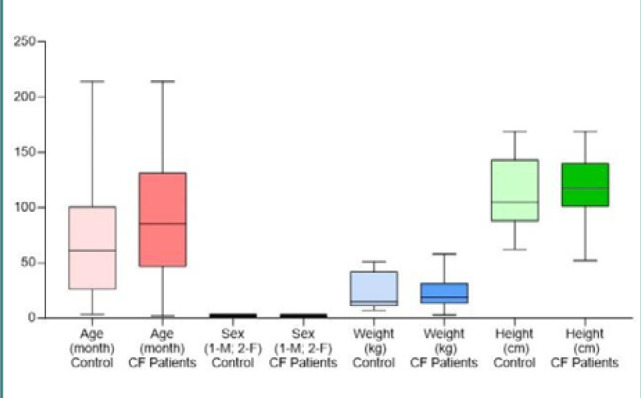
Baseline clinical characteristics of participants

**Figure 2:**
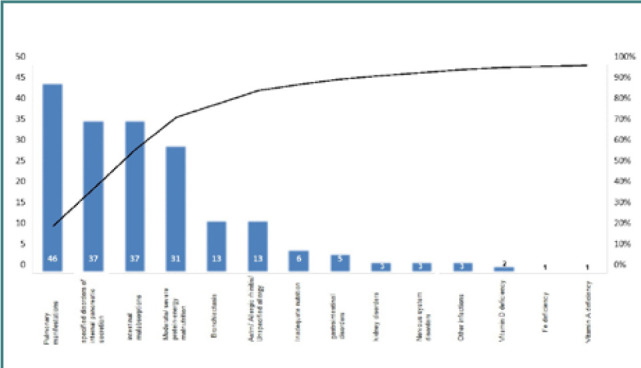
CF-patient diagnosis at the time of the study

Four (7.69%) patients were diagnosed in their first year of life. Table 1 displays the baseline clinical characteristics of these subjects. The median age of the CF group was 6.60 ± 4.30 years, compared to 4.65 ± 4.23 years in the control group. The weighted median had no significant differences between the two groups, but CF patients had a shorter average height (112.30 cm) compared to controls (118.60 cm) (Figure 1). Pulmonary symptoms were present in 88.46% of patients with CF, with no significant difference between boys and girls. What caught our attention was the prevalence of specific disorders of internal pancreatic secretion (67.30%), moderate protein-energy malnutrition (71.15%), and intestinal malabsorption (40.38%), which indicate that pediatric patients were evaluated at an advanced stage of the disease, when symptoms were too severe for parents to ignore (Figure 2).

### Biochemical assessment of liver status in patients with CF

Although elevated liver enzyme levels are commonly reported in pediatric CF patients in the literature [17], our study found no significant differences in the mean levels of AST and ALT between the CF patients and the control group (*P -* values of 0.81 for AST and 0.57 for ALT) (Table 2). However, these biochemical markers exhibit a low sensitivity (50% and 52%, respectively) and a specificity of 74% and 77%, so the observed results may not accurately assess the patient’s liver function.

### Nutritional status assessment

**Figure 3:**
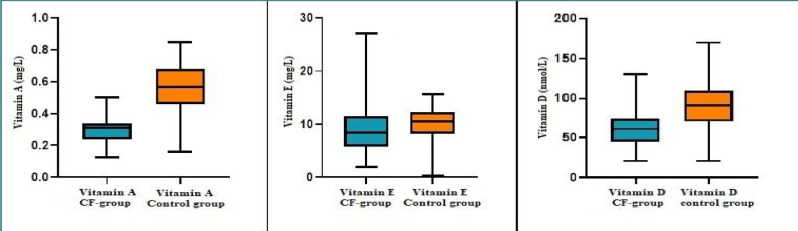
Comparison of vitamin A, E, and D levels between pediatric patients with CF and healthy controls

Given the critical importance of nutritional status in cystic fibrosis management, we analyzed and compared the levels of vitamins A, D, and E between patients with CF and healthy controls. In our cohort, only five patients with CF (9.61%) had hypovitaminosis A, with vitamin A concentrations below 0.2 mg/L. Among the patients with CF and normal vitamin A levels, 48 patients (92.30%) had concentrations below the mean level observed in the control group (Table 3). The mean vitamin A level was significantly reduced by almost two-fold, i.e., from 0.55 ± 0.15 in the control group to 0.29 ± 0.07 in the CF group (*P* < 0.05), despite the supplementation with vitamin A for patients with CF (Figure 3).

Vitamin E screening revealed that 43 pediatric patients with CF (82.70%) had a vitamin E concentration in the normal range, while two patients (3.84%) had hypovitaminosis E, and 13.46% had hypervitaminoses (Table 3). No statistically significant differences were observed between the mean vitamin E levels of patients with CF and those in the control group (Figure 3).

According to the literature, patients with CF typically have an excess of vitamin A and a vitamin E deficiency. However, our study found that the mean vitamin A levels in CF patients were nearly halved compared to the control group, whereas the mean vitamin E levels were comparable.

Regarding vitamin D, despite supplementation, only ten patients (19.23%) had vitamin D concentrations within the normal range. The remaining 42 patients (80.76%) had hypovitaminosis D, with values either suboptimal or below the detection limit (≤ 20.2 nmol/L) (Table 3). In our study, we found that 21 male patients with CF (40.38%) had vitamin D deficiency, indicating that the variation in vitamin D concentration is not dependent on gender, and there are no differences in expression level, contrary to what other researchers have shown [18]. The same tendency was observed in the control group, where 9 (52.94%) of 17 (32.69%) healthy subjects were male with vitamin D deficiency (*P* < 0.2) (Figure 3). Compared to the control group, the mean vitamin D level of the patients with CF significantly decreased by almost 67%, as seen in Table 3 (*P* < 0.05). To account for seasonal variations and avoid confounding factors such as genetic and geo-climatic influences (e.g., sunlight exposure, diet, and skin pigmentation) [18], vitamin D analysis was conducted during the cold season.

It is well known that patients with CF may be at risk for micronutrient depletion, particularly during periods of illness and infection [19]. Since 88.46% of patients with CF had pulmonary symptoms and 71.15% had moderate protein-energy malnutrition in our study, we extended our analysis to include the evaluation of serum iron (Fe), ionized calcium (Ca_i_), and total calcium (Ca_t_) levels.

**Figure 4:**
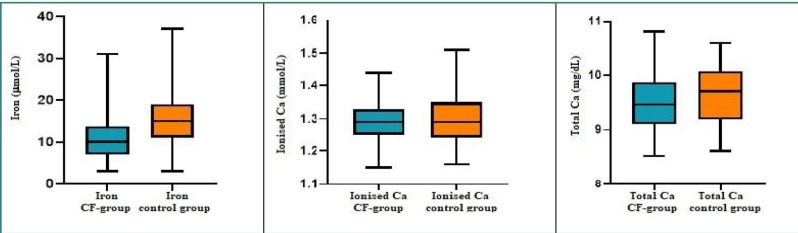
Distribution of mineral elements across the two groups

Iron screening revealed that 19 patients with CF (36.53%) had iron deficiency, and 32.6% of the patients had inadequate serum iron levels (mean level ≤ 8 µmol/L). Among the patients with CF with normal iron levels, 28 (90.32% of the 31 patients with normal Fe levels) had concentrations lower than the mean (Table 3). (While the mean iron concentration for CF patients (10.58 µmol/L) fell within the normal range, it was significantly lower compared to the mean serum iron level of the healthy control group (15.29 µmol/L) (*P* < 0.05) (Figure 4).

Contrary to our expectations, despite the vitamin D deficiency observed in patients with CF, neither ionized nor total calcium deficiencies were observed in this population. The mean Ca concentrations were similar to those of the control group (Table 3). Moreover, even though the percentage of individuals with high serum Ca levels was greater for the control group (21.15% for Ca_t_ and 19.23% for Ca_i_), 13.3% of the patients with CF (7.6% for Ca_t_ and 5.7 for Ca_i_) also had above-normal calcium levels.

### Airway bacterial colonization in patients with CF

The main symptoms of patients with CF were mucus plugging, chronic neutrophilic inflammation, and persistent poly-microbial infection of the airways by biofilm-forming gram-negative bacteria, especially *Pseudomonas aeruginosa* [19]. Activated neutrophils release various effector molecules, including neutrophil elastase, reactive oxygen species, proinflammatory cytokines, and cathepsin G, which play crucial roles in antibacterial defense but also contribute to inflammation and airway damage [19].

Analyzing hypo-pharyngeal exudates from CF patients is essential for guiding personalized therapy. In our study, we assessed bacterial colonization and found that 26 CF patients (50%) were infected with *Pseudomonas aeruginosa*, 23 patients (44.23%) with both *Pseudomonas aeruginosa* and *Burkholderia* (*pseudommalle* with or without *mallei*), 13 patients (25%) with *Methicillin-resistant Staphylococcus aureus (MRSA)/Staphylococcus aureus*. Only 14 patients (26.92%) had no bacterial colonization (Table 4).

**Table 4:** Airway bacterial colonization in patients with CF

Microorganism	Patients no.
*Pseudomonas aeruginosa*	26
*Pseudomonas aeruginosa and Burkholderia pseudommallei/mallei*	23
*MRSA/Staphylococcus aureus*	13
*Klebsiella*	1
*Candida*	1
*Proteus mirabilis and morganii*	1

**Figure 5:**
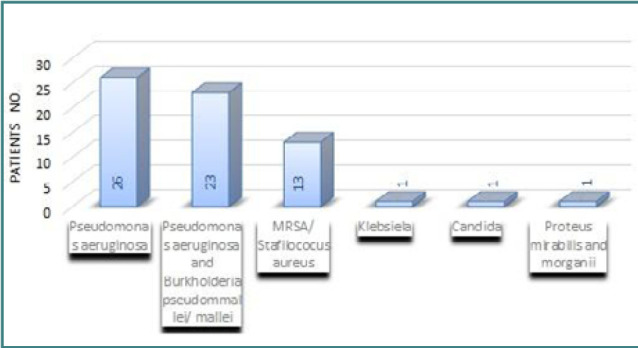
Distribution of pathogen colonization in the CF pediatric group

When correlating bacterial colonization with the clinical diagnosis at the time of the study, we observed that two patients (14.28%) were diagnosed with bronchiectasis, and eight patients (57.14%) had specific disorders of internal pancreatic secretion combined with intestinal malabsorption and moderate protein-energy malnutrition (Figure 5).

### Hematological assessment of patients with CF

**Figure 6:**
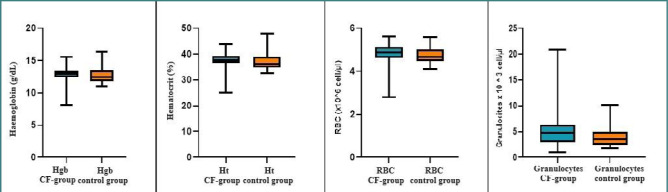
Distribution of hematologic parameters across groups

The clinical correlation between pulmonary and liver deficiencies and nutritional status in patients with CF is well-documented. However, there is limited information in the specialized literature regarding the hematological assessment of CF patients. Therefore, our study aimed to explore whether hematological changes correlate with renal and liver functions. As shown in Table 5 and Figure 6, no statistically significant differences were found between the CF and healthy control groups, except for the mean granulocyte count. The control group had an approximately 1.5-fold reduction in granulocyte number compared to the CF group (*P* < 0.05).

**Table 5:** Hematological values across study groups

Hematological parameters	CF patients Mean ± SD	Healthy patients Mean ± SD	*P* value
Hgb concentration (g/dL)	12.79 ± 1.26	12.72 ± 1.14	0.74
Ht (%)	37.33 ± 3.36	36.97 ± 3.14	0.51
Number of erythrocytes (*10^6^ cell/µl)	4.8 ± 0.49	4.7 ± 0.37	0.46
Number of granulocytes (*10^3^ cell/µl)	5.38 ± 3.5	3.97 ± 1.86	0.02

### Biochemical assessment of renal function in patients with CF

The initial assessment and ongoing monitoring of renal function in individuals are typically conducted by measuring traditional biomarkers of kidney impairment, including serum total protein, creatinine, urea, and uric acid. Accurate evaluation of renal function is crucial for appropriate drug dosing, monitoring, and early detection of kidney failure [20].

**Figure 7:**
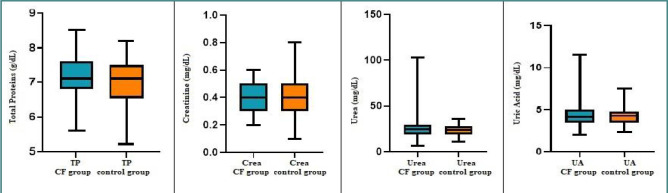
Distribution of kidney parameters across groups

In our study, the mean values of these renal biomarkers did not show any statistically significant differences between the CF and control groups. However, apart from the mean serum creatinine levels, the total protein, urea, and uric acid mean concentration levels were slightly elevated in children with CF, even if they did not reach statistical significance (Table 6). Specifically, 7.69% of children with CF had higher total protein levels, 23% exhibited hyperuricemia, and 9.6% had urea values above the normal limit (Figure 7).

**Table 6:** Kidney status across groups

Renal biomarker	CF patients Mean ± SD	Healthy patients Mean ± SD	*P* value
Total protein (g/dl)	7.19 ± 0.68	7.06 ± 0.59	0.24
Creatinine (mg/dl)	0.39 ± 0.11	0.42 ± 0.15	0.13
Urea (mg/dl)	26.46 ± 13.52	23.71 ± 6.09	0.2
Uric acid (mg/dl)	4.33 ± 1.46	4.25 ± 1.06	0.75

### Biochemical assessment of lipid status in patients with CF

It is well established that patients with pancreatic insufficiency often experience fat malabsorption, leading to disturbed lipid concentrations and an increased risk of liver cirrhosis. This condition is typically characterized by an abnormal lipid profile, including low serum cholesterol levels, due to the liver's impaired biosynthetic capacity [21]. In our study, all patients received pancreatic enzymes (Kreon or other pharmaceutical preparations) to counteract malabsorption and maldigestion. When evaluating the lipid status of patients with CF, we found that while triglyceride levels generally remained within the normal range (30-160 mg/dL for ages 0-99 years, M/F), 14 CF patients (26.92%) exhibited hypocholesterolemia, and two patients (3.84%) had elevated cholesterol levels compared to the normal range for the Romanian population The mean cholesterol (130.08 ± 30.73) and triglyceride (74.54 ± 22.15) concentrations in the entire CF cohort were statistically lower than those of the control group, regardless of age (Table 7).

**Figure 8:**
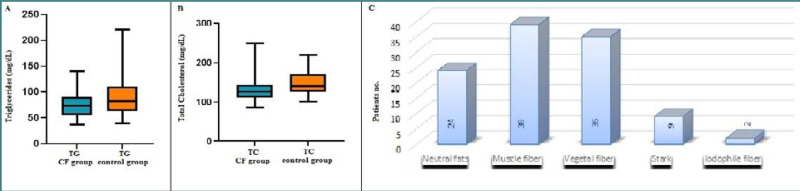
Comparison of lipid status and digestion test markers. A, B, Comparison of triglyceride (TG) and total cholesterol (TC) levels across groups; C, Distribution of specific digestion test markers (neutral fats, vegetable fibers, muscle fibers, starch, iodophile fibers) among pediatric patients with CF.

In Romania, the digestion sample test is the preferred method for diagnosing pancreatic insufficiency. Our findings showed that all CF patients had at least one positive marker on the digestion sample test (e.g., neutral fats, vegetable fibers, muscle fibers, starch, iodophile fibers), with many patients presenting two, three, or all four markers (Figure 8). This distribution is correlated with lower serum cholesterol levels, especially in children above seven years old.

**Table 7:** Lipid status across study groups

Lipids	CF patients Mean ± SD	Healthy patients Mean ± SD	*P* value
Cholesterol (mg/dL)	130.08 ± 30.73	148.52 ± 29.11	0.0008
Triglycerides (mg/dL)	74.54 ± 22.15	89.71 ± 33.99	0.01

## DISCUSSION

While CF primarily affects the respiratory system, resulting in recurring lung infections and progressive lung function decline, the condition also presents gastrointestinal manifestations and various nutritional complications. In CF, abnormal *CFTR* function in the pancreas causes the production of thick, sticky mucus, which obstructs the pancreatic ducts and impairs organ function [22]. One of the key consequences of ductal blockage is the failure to release pancreatic juice and bicarbonate into the duodenum. This results in the loss of enzymatic breakdown of nutrients in the upper bowel lumen [23]. Additionally, the low pH in the duodenum causes bile salts to precipitate, which, combined with the absence of enzymatic activity, leads to maldigestion and malabsorption of dietary fats [16]. Since the absorption of fat-soluble vitamins (A, D, E, and K) depends on the presence of fats, individuals with CF often experience deficiencies in these vitamins due to their inability to properly digest and absorb fats [24].

According to the European Society of Cystic Fibrosis (ESCF), Romania reported 311 patients with CF in 2022, of which 282 were pediatric patients [25]. Although the official incidence of confirmed CF cases in Romania has not been published, the Bucharest National Authority for 'Neonatal Screening, Diagnostic Confirmation, and Specific Treatment in CF', funded by the Romanian Ministry of Health, estimated an incidence of 1 case in 10,000 based on data collected nationwide in 2023 (unpublished results).

In this study, we analyzed the biochemical, hematological, and microbiological profiles of pediatric CF patients from a Romanian Regional Centre to assess the feasibility of clinical monitoring of life quality (LQ) and to correlate biochemical parameters with clinical manifestations.

We calculated the mean serum concentrations of vitamins A, D, and E in pediatric patients with CF and compared them to the mean levels in a healthy control group. In individuals with CF, laboratory results revealed a causal relationship between the pulmonary status of patients and their vitamin A concentrations, an observation that suggests that vitamin A may have a protective effect on the pulmonary status of individuals [26]. In our study, the vitamin A levels in children with CF were reduced by half compared to those in the healthy control group, despite supplementation. However, less than 10% of the children with CF had vitamin A below the reference limit, consistent with other studies that report vitamin A deficiency as relatively uncommon among patients with CF [10,14,16,22].

Another essential nutrient necessary for proper metabolic function is vitamin D, a fat-soluble molecule with an indispensable role in Ca homeostasis, immunity, and infection prevention [27]. Vitamin D deficiency is often associated with skeletal complications and an increased risk of infections [28]. It is well established in the literature that approximately 90% of individuals diagnosed with CF experience pancreatic insufficiency, necessitating additional vitamin D supplementation. However, despite this, many patients remain vitamin D deficient, often due to a combination of factors such as fat malabsorption, impaired hydroxylation of vitamin D, decreased sun exposure, and non-adherence to the prescribed diet [10,14,16,29]. Our study demonstrated that, unlike vitamin A, where only a small number of children had hypovitaminosis A, the prevalence of vitamin D deficiency in the CF group was significantly higher, with 42 children showing suboptimal or below-limit vitamin D levels despite supplementation. These findings were expected and corroborate data reported in the literature [30].

Vitamin E, known for its antioxidant properties, helps protect the body against oxidative stress, which can lead to irreversible cellular damage. Moreover, vitamin E also improves the health of red blood cells, combats infections, and helps maintain proper intestinal function [29,30]. In patients with cystic fibrosis, low levels of vitamin E can cause anemia or a decrease in red blood cell count, making supplementation necessary [10,14,16]. Contrary to other research, in the present study, vitamin E deficiency was less common, with 10% of patients exhibiting above-normal vitamin E levels. However, available studies reported that the risk of vitamin E deficiency increases during inflammation, particularly in the digestive and respiratory systems [31].

In contrast to the focus on fat-soluble vitamins, the status of other nutrients, such as minerals, has received less attention, primarily due to the lower prevalence of reported deficiencies. In this study, we analyzed the mean levels of iron, ionized calcium, and total calcium in the serum of pediatric CF patients and compared them to those of healthy controls.

Iron deficiency is frequently reported in individuals with CF, especially those with advanced lung disease, due to chronic inflammation, malnutrition, impaired dietary iron absorption, and loss of iron through sputum [32]. The high incidence of hypoferremia in patients with an exacerbated lung condition could be attributed to their Fe homeostasis being tightly associated with *Pseudomonas aeruginosa* colonization - an essential factor in the evolution of their respiratory condition [33]. Moreover, there is a growing fear that Fe supplementation may be responsible for promoting *Pseudomonas aeruginosa*-associated infections since it has been demonstrated to increase bacterial growth in vitro [34]. Our study revealed an increased Fe level in more than half of patients with CF, above the recognized threshold but still reduced compared to the mean iron serum levels in the healthy control group. Furthermore, we observed an association between Fe levels and pathogenic colonization, i.e., no bacterial colonization was observed in children with CF and Fe levels similar to those of the age-matched control group. This finding suggests that recurrent bacterial lung infections may lead to iron loss through sputum, contributing to iron deficiency. Additionally, in CF patients with bacterial colonization, we observed a nearly 1.5-fold increase in granulocyte count, possibly due to the impact of bacterial infection on granulocyte count [35]. It is well known that there is a direct interdependence between vitamin A and iron levels. Consequently, patients with hypovitaminosis A may also have reduced iron levels, as vitamin A plays a crucial role in erythropoiesis [36].

Calcium is another essential element with pivotal roles in bone homeostasis, the normal functioning of various enzymes, neuronal transmission, myocardial function, blood coagulation, and other cellular functions [37]. Our study observed a negative Ca balance in patients with CF, likely due to the high prevalence of vitamin D deficiency caused by malabsorption and possibly altered intestinal permeability [13,16,24]. In contrast, calcium was one of the most abundant elements in the control group. To evaluate the calcium status in both groups, we assessed the levels of ionized and total calcium. The mean serum levels were above the limit values, with a slight elevation in the case of total Ca levels. However, the evaluation of total Ca can be a misleading marker since ionized Ca is biologically active and is tightly regulated by Ca-binding hormones. Therefore, ionized Ca is a more helpful maker than total Ca and can better indicate the patient's Ca status.

Due to the poor control of malabsorption and subsequent energy loss, individuals with CF are often prescribed energy-dense diets based on high-fat foods, which in a healthy population are known to be a risk factor for a series of cardiovascular diseases [16,38]. However, regardless of the prescribed diet, the mean cholesterol level was statistically lower than that of the healthy control group. Furthermore, regardless of age, it was observed that in the case of patients with pancreatic insufficiency, hypocholesterolemia was more frequent. In contrast, the concentrations of triglycerides were not affected by this condition. Altogether, the lipid status observed in our screening study agrees with other studies that report abnormal lipid profiles in CF patients compared to healthy individuals.

With increased life expectancy and improved medical care quality, many complications, such as kidney injury, which previously raised little concern, have become important to clinicians [39]. Although the kidneys are not directly affected by CF, they can be impacted later in life due to aggressive treatments for lung infections. Individuals with CF present a greater risk of developing acute kidney injury caused either by the potentially nephrotoxic medication (e.g., aminoglycosides, colistin, non-steroidal anti-inflammatory drugs) or by systemic chronic inflammation and diabetes [40, 41]. Acute kidney injury, formerly known as acute renal failure, is defined in the specialized literature as an acute but reversible condition in which an increase in various waste products (e.g., creatinine, ammonia, urea, and uric acid) is observed in combination with the kidney’s inability to regulate electrolytes and fluids [42]. Our study did not find any statistically significant differences in the renal status of patients with CF compared to the healthy control group. However, it is worth mentioning that despite the lack of statistical significance, children with CF had slightly elevated mean serum concentrations of total protein and uric acid. A possible explanation could be the recurrent infection, chronic inflammation, and pancreatic enzyme supplementation (which contains high levels of purines).

Additionally, the elevated urea levels and suboptimal serum creatinine levels observed in our study may suggest impaired renal function in the screened CF patients. However, serum creatinine is an unreliable marker of kidney function in patients with CF due to their reduced muscle mass, often leading to overestimating actual renal function [20].

Hepatic abnormalities are also common in the early stages of CF, particularly affecting children and adolescents [43]. While the pathophysiology of the condition is largely unknown, its clinical expression includes hepatic steatosis, portal hypertension, biliary fibrosis, and, finally, multi-lobar cirrhosis [44], an affliction associated with gastrointestinal bleeding, malnutrition, and ascites. AST and ALT levels are commonly used to assess liver function in patients with CF. Whereas AST is less specific for liver injury even though it is found in high concentrations in the liver, ALT is found primarily in hepatocytes, and its abnormal concentrations can indicate a parenchymal liver condition characterized by varying degrees of necrosis [43]. As such, routine assessment for liver disease is recommended in people with CF, including annual blood tests and regular ultrasonography [45]. People with CF have naturally fluctuating liver transaminase levels. Persistently elevated transaminase levels (greater than 3 times the upper limit of normal) and abnormal clinical findings should prompt an abdominal ultrasound. Contrary to the data reported in the literature, no statistically significant differences between the mean values of AST and ALT were observed in the present study, suggesting that the liver function of the screened patients has not yet been compromised. However, as previously mentioned, AST and ALT are not the most reliable indicators of liver function because they lack both sensitivity and specificity [46]. Additionally, the upper threshold for ALT varies significantly between clinics and is often set too high to effectively detect liver disease in children.

## CONCLUSION

Clinical analysis of biochemical status has long been used to monitor the biochemical parameters of patients with CF and to guide pediatric physicians in providing personalized therapeutic approaches. However, no studies have been conducted on pediatric patients in Romania. Previous research in the country has primarily focused on the progress and quality of life of CF patients over 18. The present pilot study is the first in Romania and will facilitate long-term research on CF within the country. It will also contribute to developing and regularly updating national guidelines for monitoring and treating patients with CF.

In conclusion, the pediatric patients diagnosed with CF screened in this study had laboratory values outside the normal range, with significant variability regardless of age. The children in this study displayed deficiencies in vitamins A and D, iron, and alterations in lipid status. It is important to note that infants and children are a unique population segment with a more active and constantly changing metabolism than adults. Therefore, further research is needed to better predict the course of their disease and develop treatment plans tailored to the individual needs of each patient.

## Personal thanks

'National Program for Women's and Child Health (XIII), Subprogram 1, Nutrition and Child Health, Intervention 1.2.1, Prevention of associated morbidity and complications, through early diagnosis, as well as monitoring of chronic diseases in children, 1.2.1.3. -Mucoviscidosis in a child' and 'National Program for Women's and Child Health (XIII), Sub-programme 1, Nutrition and child health, intervention 1.3.5. -Dietary treatment of children with cystic fibrosis'. We also want to acknowledge the support of the CNFIS-FDI-2024-F-0498 INOVEX project: 'Support in the Development of new competence and innovative tools for excellence in interdisciplinary research at the University of Bucharest'.
