# Supplementary material for: Biochemical assessment in a cohort of pediatric patients with cystic fibrosis
Source: J Med Life. 2024 Jun;17(6):610–9. doi: 10.25122/jml-2024-0288 (PMC11407493; doi:10.25122/jml-2024-0288)
Supplement: Supplementary file 1 [file JMedLife-17-610-s001.pdf]

Table 1S. Normal values of biochemical parameters, depending on age and sex, in the Romanian population

AST (UI/L)	ALT (UI/L)	Vitamin A (mg/L)	Vitamin E (mg/L)	Vitamin D (nmol/L)	Serum iron (µmol/L)	Ionised calcium (mmol/L)	Total calcium (mg/dL)
2-6 y: 10-50<br>≥7 y: 10-37	≥1 y: 10-60	0.2–0.8 mg/L	3–14 mg/L	75–175 nmol/L	5–160 d:6–28<br>6 m-13 y: 8–31<br>>13 y: 11–32 (M)<br>>13 y: 8-28 (F)	1.12–1.35	8,6–10,1
Hgb (g/dL)	Ht (%)	Erythrocytes no. (*10^6 cell/µl)	Granulocytes no. (*10^3 cell/µl)	Total protein (g/dl)	Creatinine (mg/dl)	Cholesterol (mg/dL)	
2-3 mo: 10.0-13.0<br>4-6 mo: 10.5-14.3<br>6-8 mo: 10.5-14.3<br>8-12 mo: 11.0-14.5<br>1-2 y: 11.0-15.0<br>3-6 y: 12.0-15.0<br>7-12 y: 12.5-15.0<br>13-18 y: 13.0-15.0	2-3 mo: 36-45<br>4-6 mo: 36-44<br>6-8 mo: 36-45<br>8-12 mo: 36-45<br>12-18 y: 39-45	2-3 mo: 3,5 - 4,5<br>4-6 mo: 4,0 - 5,0<br>6-8 mo: 4,0 - 5,0<br>8-12 mo: 4,1 - 5,1<br>1-2 y: 4,0 - 4,6<br>3-6 y: 4,2 - 5,2<br>7-12 y: 4,5 - 5,4<br>13-18 y: 4,5 -5,4	2-3 mo: 1.8-5.4<br>4-6 mo: 1.2-7.5<br>6-8 mo: 1.2-8.5<br>8-12 month: 1-8.5<br>1-2 y: 1-8.5<br>3-18 y: 1.5-8.5	2 mo -1 y: 4.8-7.4<br>1-12 y:6.2-8.0<br>13-18 y:6.4-8.4	0-13 y: 0.2-1<br>>14 y: 0.4-1.4	29 d-1 y: 60 – 190<br>1-4 y: 95 – 190<br>5-12 y: 110 – 190<br>13-18 y: 140-200	
Triglycerides (mg/dL)	Urea (mg/dl)	Uric acid (mg/dl)					
30 -160	1-17 y: 15-35<br>>18 y: 3.5-7.2	<18 y: 2-5<br>≥19 y: 3.5-7.2 (M)<br>≥19 y: 2.4-6.0 (F)					

Abbreviations: d-days; mo-month; y-years; M- male; F-female

Table 2S. The screened patients' genotype

No.	Patient sex	Patient genotype
1	M	DF508/DF508
2	M	DF508/DF508
3	M	DF508/DF508
4	M	F508/c.325_delTATTinsG
5	F	W128X/C1894_1895delGA
6	F	DF508/C1646G >T
7	M	DF508/DF508
8	M	DF508/DF508
9	F	DF508/DF508
10	F	DF508/R1066C
11	M	G524X/c.3758T >C
12	F	DF508/ c.14C >T
13	F	DF508/DF508
14	M	DF508/DF508
15	M	DF508/X
16	F	DF508/X
17	F	F508/ c3758T >C
18	M	DF508/DF508
19	F	DF508/DF508
20	M	F508del:1677delTA
21	M	F508del,G542X
22	M	G85E/S549N7T
23	F	DF508/DF508
24	M	G126D/3040+1G >A
25	M	CFTRdel2,3/C276X
26	M	DF508/DF508

No.	Patient sex	Patient genotype
27	F	c.828C>Ap.CYS276*/ c.205delAp. Lys684Asnf*38
28	F	DF508/DF508
29	M	DF508/X
30	F	DF508/DF508
31	M	DF508/DF508
32	F	DF508/X
33	F	DF508/DF508
34	F	DF508/DF508
35	F	DF508/DF508
36	M	DF508/X
37	F	DF508/X
38	F	DF508/DF508
39	F	DF508/DF508
40	M	F508del si Gly542X
41	M	DF508/DF508
42	M	DF508/DF508
43	M	DF508/W1282X
44	F	DF508/X
45	M	7T/9T
46	F	G551/DF508
47	F	G551/DF508
48	M	DF508/E882X
49	F	DF508/L88X/G1069R
50	M	DF508/DF508
51	M	DF508/DF508
52	M	DF508/X

Abbreviations: M- male; F- female

© 2024 by the authors. This article is an open access article distributed under the terms and conditions of the Creative Commons Attribution (CC BY 4.0) license.

Table 3S. Distribution of a genotype among patients with CF

Patient genotype	Percentage of patients with a specific genotype
DF508/DF508	24 (46.1%)
DF508/X	8 (15.3%)
DF508/C1646G >T	1 (1.9%)
F508/c.325_delTATTinsG	1 (1.9%)
DF508/R1066C	1 (1.9%)
G524X/c.3758T >C	1 (1.9%)
DF508/c.14C >T	1 (1.9%)
F508/ c3758T >C	1(1.9%)
F508del:1677delTA	1 (1.9%)
F508del,G542x	1 (1.9%)
G85E/S549N7T	1 (1.9%)
G126D/3040+1G>A	1 (1.9%)
CFTRdel2,3/C276X	1 (1.9%)
c.828C>As.Cys276*/c.2052delAp.Lys684Asnfs*38	1 (1.9%)
F508del si Gly542X	1 (1.9%)
DF508/W1282X	1 (1.9%)
7T/9T	1 (1.9%)
G551/DF508	2 (3.8%)
DF508/E882X	1 (1.9%)
DF508/L88X/G1069R	1 (1.9%)
W128X/C1894_1895delGA	1 (1.9%)

© 2024 by the authors. This article is an open access article distributed under the terms and conditions of the Creative Commons Attribution (CC BY 4.0) license.
